# Dopamine imbalance in Huntington's disease: a mechanism for the lack of behavioral flexibility

**DOI:** 10.3389/fnins.2013.00114

**Published:** 2013-07-04

**Authors:** Jane Y. Chen, Elizabeth A. Wang, Carlos Cepeda, Michael S. Levine

**Affiliations:** Intellectual and Developmental Disabilities Research Center, Semel Institute for Neuroscience and Human Behavior and the Brain Research Institute, David Geffen School of Medicine, University of California Los AngelesLos Angeles, CA, USA

**Keywords:** Huntington's disease, behavioral inflexibility, dopamine, glutamate, electrophysiology

## Abstract

Dopamine (DA) plays an essential role in the control of coordinated movements. Alterations in DA balance in the striatum lead to pathological conditions such as Parkinson's and Huntington's diseases (HD). HD is a progressive, invariably fatal neurodegenerative disease caused by a genetic mutation producing an expansion of glutamine repeats and is characterized by abnormal dance-like movements (chorea). The principal pathology is the loss of striatal and cortical projection neurons. Changes in brain DA content and receptor number contribute to abnormal movements and cognitive deficits in HD. In particular, during the early hyperkinetic stage of HD, DA levels are increased whereas expression of DA receptors is reduced. In contrast, in the late akinetic stage, DA levels are significantly decreased and resemble those of a Parkinsonian state. Time-dependent changes in DA transmission parallel biphasic changes in glutamate synaptic transmission and may enhance alterations in glutamate receptor-mediated synaptic activity. In this review, we focus on neuronal electrophysiological mechanisms that may lead to some of the motor and cognitive symptoms of HD and how they relate to dysfunction in DA neurotransmission. Based on clinical and experimental findings, we propose that some of the behavioral alterations in HD, including reduced behavioral flexibility, may be caused by altered DA modulatory function. Thus, restoring DA balance alone or in conjunction with glutamate receptor antagonists could be a viable therapeutic approach.

## Introduction

Huntington's disease (HD) is an inherited, autosomal dominant, and progressive neurodegenerative disorder caused by a mutation in the huntingtin gene (*HTT*) resulting in an abnormally long polyglutamine (CAG >40) repeat (The Huntington's Disease Collaborative Research Group, 1993). It is characterized by involuntary dance-like movements (chorea) in the early stages, then akinesia and dystonia in the late stages. Other symptoms include psychiatric alterations and cognitive deterioration (Bonelli and Hofmann, 2007). Cognitive disturbances affecting learning, memory processes, as well as attention and executive function emerge early in the course of the disease and become prominent in the advanced stages (Brandt and Butters, 1986; Peinemann et al., 2005; Wang et al., 2012). A juvenile form of HD also occurs, generally when the length of CAG repeats is >60. These patients develop epileptic seizures and intellectual decline associated with a more rapidly progressing course of the disease (Andrew et al., 1993; Seneca et al., 2004).

In HD, the most striking neuropathology is massive loss of medium-sized spiny neurons (MSNs) in the striatum (Vonsattel and Difiglia, 1998), as well as laminar thinning and white matter loss in the cerebral cortex (Rosas et al., 2006). Other structures such as the globus pallidus, thalamus, hypothalamus, subthalamic nucleus (STN), and substantia nigra also are affected, particularly in the later stages (Kremer et al., 1990; Heinsen et al., 1996; Petersen et al., 2005). Although the symptomatology of HD is classically attributed to striatal and cortical neuronal loss, studies have demonstrated that neuronal dysfunction precedes cell death (Tobin and Signer, 2000; Levine et al., 2004). For example, psychiatric, cognitive, and motor symptoms can and often appear alongside cellular and synaptic alterations in the absence of neuronal loss (Vonsattel and Difiglia, 1998).

This review examines the role of striatal dopamine (DA) in HD. We focus on neuronal electrophysiological mechanisms that may lead to some of the motor and cognitive symptoms of HD and how they relate to dysfunction in DA neurotransmission. Data from human and animal studies are reviewed with particular emphasis on alterations of the DA system and how they relate to behavioral inflexibility. The central thesis is that the major symptoms of HD can be associated with biphasic changes in DA transmission and its modulatory role on glutamate (GLU) receptor function. Thus, treatments of HD symptoms should take into account and be tailored according to the temporal progression of neurotransmitter and receptor changes. Before elaborating on these changes, we first need to understand the role of the DA system and its interactions in normal neuronal function, particularly in the striatum.

## Striatal organization

**Figure 1:**
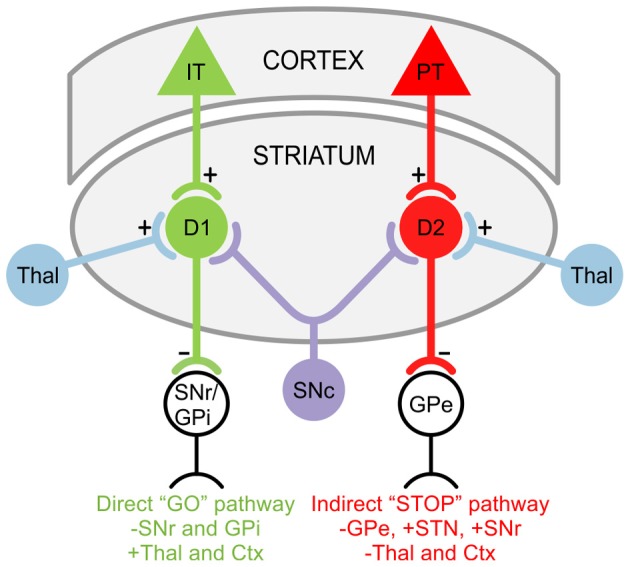
**Striatal projection pathways**. In the direct “GO” pathway, MSNs expressing DA D1 receptors receive inputs from intratelencephalically projecting (IT) neurons in the cortex (Ctx) and project to the substantia nigra pars reticulata (SNr) as well as the internal segment of the globus pallidus (GPi). In the indirect “STOP” pathway, MSNs expressing DA D2 receptors receive inputs from pyramidal tract (PT) neurons in the Ctx and project to the external segment of the globus pallidus (GPe). The GPe, in turn, projects to the STN and SNr. Both D1 and D2 MSNs also receive afferents from the substantia nigra pars compacta (SNc) and thalamus (Thal).

GABAergic projection MSNs comprise 90–95% of striatal neurons (Kita and Kitai, 1988) and receive glutamatergic inputs primarily from the cortex as well as specific thalamic nuclei (Kemp and Powell, 1971; Smith et al., 2004). There are two striatal projection pathways (Figure 1), each with distinct MSN populations expressing different DA receptors and neuropeptides (Graybiel, 2000). The direct pathway consists of MSNs expressing DA D1 receptors, substance P, and dynorphin (Vincent et al., 1982; Haber and Nauta, 1983; Gerfen et al., 1990). It projects monosynaptically to the substantia nigra pars reticulata and the internal segment of the globus pallidus (Albin et al., 1989; Gerfen et al., 1990). The indirect pathway is composed of MSNs that express D2 receptors, adenosine A_2A_ receptors, and enkephalin (Gerfen et al., 1990; Schiffmann and Vanderhaeghen, 1993; Steiner and Gerfen, 1999), and projects to the external segment of the globus pallidus (Gerfen, 1992; Bolam et al., 2000). The external segment of the globus pallidus, in turn, projects to the STN (Albin et al., 1989). Electrophysiological studies using mice expressing enhanced green fluorescent protein (EGFP) in MSNs enriched with D1 or D2 DA receptors demonstrated that, although direct and indirect pathway neurons display similar basic membrane properties, indirect pathway MSNs are more excitable and thus may be more susceptible to abnormal GLU release or receptor dysfunction (Kreitzer and Malenka, 2007; Cepeda et al., 2008). This is partially due to a difference in dendritic surface area, where indirect pathway MSNs have fewer primary dendrites than direct pathway MSNs, suggesting that the increased excitability of indirect pathway MSNs partially results from a higher membrane input resistance due to their more compact morphology (Gertler et al., 2008; Flores-Barrera et al., 2010). The remaining 5–10% of striatal neurons are interneurons, which are divided into two main groups: GABAergic interneurons, which provide feedforward inhibition to MSNs (Tepper et al., 2008); and cholinergic interneurons, which are responsible for acetylcholine levels in the striatum (Bolam et al., 1984; Zhou et al., 2002).

The striatum can also be described as a mosaic of two functionally distinct compartments. The striosome compartment is enriched with μ-opioid receptors while the surrounding extrastriosomal matrix contains neurons that express acetylcholinesterase, somatostatin, and calbindin (Gerfen, 1984). GABAergic striosomal neurons innervate DA neurons in the substantia nigra pars compacta and reticulata, essentially forming a third striatal output pathway (Gerfen, 1984; Jimenez-Castellanos and Graybiel, 1989). Since interactions between striosomes and the extrastriosomal matrix are involved in drug-induced stereotypies (Saka et al., 2004; Canales, 2005), it has been proposed that the striosomal system may change the set point of DA neurons (Canales and Graybiel, 2000). This, in turn, could modulate DA neurotransmission in the basal ganglia and alter the occurrence of stereotypic behaviors (Graybiel, 2000). As discussed later, pathological changes in the striosome compartment could underlie dysregulation of DA release in the early stages of HD.

## Modulatory role of DA in the brain

The modulatory effects of DA are better understood if considered as a representation of an inverted “U” shaped function. This concept suggests that too much or too little DA perturbs cognitive function (Williams and Castner, 2006; Vijayraghavan et al., 2007). Furthermore, maximum efficiency in behavioral and cognitive performance is a result of maintaining an optimal DA level, where imbalances cause decreased efficiency (Dickinson and Elvevag, 2009). As an extension, we can say that in the dorsal striatum, increases or decreases in DA alter motor behavior.

One of the main functions of DA in the brain is to enhance the signal-to-noise ratio. This can be achieved by at least 3 different mechanisms: (1) DA can modulate neuronal firing in a selective manner. For example, studies in awake rats show that iontophoresis of DA induces excitation of motor-related, and inhibition of non-motor-related neurons (Pierce and Rebec, 1995). Also, the effect of D1 agonists on neuronal firing can be excitatory or inhibitory depending on the membrane potential of the cell. At hyperpolarized potentials, D1 receptor activation is inhibitory, whereas at depolarized potentials, it is excitatory (Hernandez-Lopez et al., 1997). (2) DA affects responses evoked by GLU in a differential manner. Responses evoked by activation of α-amino-3-hydroxyl-5-methyl-4-isoxazole-propionic acid (AMPA) receptors are reduced by DA, whereas responses evoked by activation of N-methyl-D-aspartate (NMDA) receptors are increased by DA (Cepeda et al., 1993; Levine et al., 1996; Cepeda and Levine, 1998). In general, activation of D1 receptors enhances GLU responses whereas activation of D2 receptors decreases these responses (Cepeda et al., 1993). (3) DA also can select excitatory inputs to the striatum (Flores-Hernandez et al., 1997) and thus act as a filter for less active inputs (Bamford et al., 2004). These effects are probably mediated by presynaptic D2 receptors located on corticostriatal GLU terminals (Cepeda et al., 2001). DA modulation of neurotransmitter release also is influenced by endocannabinoid production and retrograde activation of presynaptic corticostriatal CB1 receptors (Maejima et al., 2001; Patel et al., 2003; Kreitzer and Malenka, 2005).

## DA and behavioral inflexibility

Behavioral inflexibility is defined as a failure to shift between behaviors and the inability to adapt behavior to changes in environmental stimuli. Lack of behavioral flexibility depends on the inability to stop ongoing behaviors and is mediated by a discrete cortico-basal ganglia circuit (Aron and Poldrack, 2006; Aron et al., 2007). Although behavioral routines are often stereotyped through learning and result in habit formation, extremely repetitive behaviors (stereotypies) appear to be prominent symptoms in various neuropsychiatric disorders and addiction. These range from impaired behavioral inhibition in attention deficit/hyperactivity disorder and inability to suppress emotions in autism spectrum disorders, to repetitive twitches or vocalizations in Tourette's syndrome, movement fixation in obsessive-compulsive disorder, punding due to over-medication of Parkinson's disease patients, and may include some of the involuntary movements in HD. Despite the wide range of behavioral phenotypes in these disorders, central features of these behaviors are DA-dependent and related to striatal dysfunction (Frank et al., 2004; Beste et al., 2010).

Changes in the DA system have long been implicated in human cognitive inflexibility. However, patients with DA impairments do not show deficits on all tasks that assess cognitive flexibility. Specifically, DA function in the striatum involves set-shifting between object features but is not involved in shifting between abstract rules (Cools et al., 2006; Dang et al., 2012). Patients with disorders of the basal ganglia, such as in Parkinson's Disease or HD, routinely show cognitive inflexibility as demonstrated by impaired performance on the Wisconsin Card Sorting Test and attentional set-shifting tests (Owen et al., 1993; Lawrence et al., 1996).

In the striatum, different subregions are involved in specific behavioral strategies and learning. Rats with lesions of the lateral striatum have deficits in motor skill learning and arbitrary stimulus-response associations (Reading et al., 1991; Devan et al., 1999), whereas those with lesions of the medial striatum have impairments in spatial and reversal learning (Whishaw et al., 1987; Pisa and Cyr, 1990). Furthermore, the medial striatum plays a role in switching between navigational strategies in response to changes in the environment (Mizumori et al., 2000). The dorsomedial striatum also is necessary for maintaining and executing a new strategy. Failure to maintain a proper response pattern by shifting strategies results in behavioral inflexibility (Ragozzino, 2007). Additionally, reversal learning and trait impulsivity in mice is associated with DA receptor density in the midbrain (Dalley et al., 2007; Lee et al., 2009). Taken together, these studies indicate that the striatum and DA neurotransmission play a crucial role in determining behavioral flexibility.

Stereotypies in rodents are an extreme form of behavioral inflexibility that manifest as rigid, repetitive movements. These include excessive grooming, sniffing, rearing, as well as locomotion, and may be more manifest during social isolation and stress (Ridley, 1994). Stereotypies present as behavioral abnormalities with little flexibility and high repetition, often similar to addictive states. Drugs that act on the DA system can produce stereotyped behaviors in a dose-dependent manner. For example, low doses of amphetamine and cocaine induce repetitive locomotion while high doses cause more focal stereotypy, such as sniffing and grooming (Cooper and Dourish, 1990). Striatal cocaine administration also results in impaired reversal learning (Stalnaker et al., 2009), further indicating that aberrant DA transmission results in behavioral inflexibility. The intensity of drug-induced stereotypies is determined by striatal DA, where rats with high extracellular DA levels demonstrate complex stereotypic behavior, including syntactic grooming (Berridge et al., 2005). In fact, robust stereotypies in rats similar to those induced by amphetamine and cocaine can be induced by striatal infusions of D1 and D2 receptor agonists (Waszczak et al., 2002).

It would be misleading, however, to think that only DA alterations are involved in behavioral inflexibility. In fact, the capacity for attentional shifts and inhibition of ongoing motor activity by salient stimuli seems to depend on thalamostriatal inputs onto cholinergic interneurons (Ding et al., 2010). These aspiny interneurons have rich terminal connections and are implicated in stereotypic behavior as well as associative learning (Aosaki et al., 1994, 2010). For example, striatal application of the muscarinic receptor antagonist pirenzepine impairs reversal learning, indicating that these cholinergic receptors play a role in the shifting of response patterns (Tzavos et al., 2004). Thus, cholinergic interneurons may also play an important role in the loss of cognitive and behavioral flexibility in pathological conditions including HD.

## DA alterations in huntington's disease

Alterations in DA function play a significant role in the motor and cognitive symptoms of HD. Here we will discuss changes in DA transmission that may underlie the neuropathological changes in HD. There is evidence from studies in HD patients that increased DA release induces chorea while a reduction in DA leads to akinesia (Bird, 1980; Spokes, 1980), thus giving rise to the biphasic movement symptoms of early and late HD. The idea that aberrant DA signaling underlies behavioral abnormalities was first proposed as a predictive test when asymptomatic offspring of individuals with HD developed dyskinesias in response to levodopa (L-DOPA) administration (Klawans et al., 1970). The hypothesis was that stimulation of DA receptors was involved in the production of dyskinesias as a basic mechanism of chorea. Early studies indicating an involvement of the DA nigrostriatal pathway in HD demonstrated increased levels of DA in postmortem brains of HD patients and showed that DA-depleting agents and DA receptor agonists can be used with therapeutic benefit (Bird, 1980; Spokes, 1980). Later, neurochemical studies of HD patients suggested that increased DA occurs in the early stages of the disease (Garrett and Soares-Da-Silva, 1992) while postmortem studies of late-stage HD patients showed reduced levels of caudate DA and homovanillic acid, the principal DA metabolite (Bernheimer et al., 1973; Kish et al., 1987). Thus, it was thought that DA levels in HD may show biphasic, time-dependent changes, with early increases followed by late decreases (Table 1).

**Table 1:** **DA in human HD and animal models**.

	**Early stage**	**Late stage**
**HUMAN HD**		
DA levels in striatum	Increased	Decreased
	Garrett and Soares-Da-Silva, 1992	Bernheimer et al., 1973; Kish et al., 1987
DA receptor density	Decreased	Decreased
	Joyce et al., 1988; Richfield et al., 1991; Van Oostrom et al., 2009	Antonini et al., 1996; Weeks et al., 1996
DAT	Not determined	Decreased
		Backman et al., 1997; Ginovart et al., 1997; Suzuki et al., 2001
**ANIMAL MODELS**		
DA levels	Increased^*^	Decreased
	^*^tgHD rat model	Hickey et al., 2002; Johnson et al., 2006; Callahan and Abercrombie, 2011
	Jahanshahi et al., 2010	
DA receptors	Decreased	Decreased
	Cha et al., 1998; Bibb et al., 2000; Ariano et al., 2002; Petersen et al., 2002b	Pouladi et al., 2012
DAT	Not determined	Not determined

During the early phase of HD, neuropathological studies have shown that discrete islands of neuronal loss and astrocytosis appear in the striosomes almost exclusively, whereas in the late phase, cell loss increasingly occurs in the matrix compartment (Hedreen and Folstein, 1995). As MSNs from the striosomes project to the substantia nigra pars compacta, it may be that early degeneration of these inhibitory neurons produces hyperactivity of the DA pathway, contributing to chorea and other early clinical manifestations of HD. Studies using positron emission tomography, autoradiography, and markers for pre- and postsynaptic neurons have observed reduced striatal D1 and D2 DA receptor density, even in asymptomatic HD patients, further indicating that DA signaling is disrupted early in HD (Joyce et al., 1988; Richfield et al., 1991; Van Oostrom et al., 2009). These observations have been confirmed by imaging studies, which reported reduced striatal D1 and D2 receptors in both HD patients and asymptomatic HD mutation carriers (Antonini et al., 1996; Weeks et al., 1996). There also is a progressive reduction of D1 and D2 receptor binding in the temporal and frontal cortices (Ginovart et al., 1997; Pavese et al., 2003). Striatal and cortical loss of DA receptors in presymptomatic and early stage HD patients have been correlated with early cognitive decline, which may reflect altered synaptic plasticity and lead to deficits in cognitive processes such as attention, executive function, learning, and memory (Backman and Farde, 2001).

Studies also have examined DA transporter (DAT) density as both an index of DA neurotransmission and a correlate of clinical status (Hwang and Yao, 2011). DAT is a key regulator of DA receptor stimulation and, in turn, affects locomotion and cognitive function. DA transmission is initiated by DA release from the presynaptic terminal and is terminated by its reuptake through DAT. In fact, postmortem analyses of brains from HD patients have shown reduced striatal DAT binding and reduced levels of vesicular monoamine transporter type-2, which is used to estimate the extent of DA innervation (Backman et al., 1997; Ginovart et al., 1997; Suzuki et al., 2001). This indicates that the reduction in DAT binding likely results from a loss of DA nigrostriatal terminals, consistent with the view that the dystonic late-stage symptoms of HD may arise in part from critical reductions in DA input.

## DA in animal models of HD

Animal models of HD have been available for more than 30 years, beginning with the first neurotoxin-based models in which chemically-induced striatal lesions reproduced HD neuropathology, providing insights into the mechanisms underlying striatal cell death (Difiglia, 1990; Brouillet et al., 1999). After the discovery of the HD gene, transgenic and knock-in rodent models were generated. These better replicated the processes and mechanisms underlying the slow development of the human disease far beyond endpoint analyses. We have previously reviewed the phenotypic properties of a number of these models (Cepeda et al., 2010; Raymond et al., 2011). Here, we will briefly describe those that have been used for electrophysiological studies examining DA neurotransmission.

The most widely used mouse model for electrophysiology is the R6/2 line, a transgenic fragment model expressing exon 1 of *HTT* with ~150 CAG repeats (Mangiarini et al., 1996). R6/2 mice display a very rapidly progressing phenotype, similar to the juvenile form of HD in humans. In these mice, overt symptoms begin to appear at 5–7 weeks of age and become fully manifest after 8 weeks. The R6/1 transgenic mouse model, with ~110 CAG repeats and less mutant *HTT* expression than the R6/2, displays similar phenotypic alterations but in a more protracted form (Mangiarini et al., 1996). HD mouse models with full-length mutant *HTT* include the yeast artificial chromosome model with 128 CAG repeats (YAC128) and the bacterial artificial chromosome model with 97 CAG/CAA repeats (BACHD) (Slow et al., 2003; Gray et al., 2008). These models show a longer development of the HD phenotype and thus are generally studied at the early (1.5–2 months of age) and late stages (12 months of age), corresponding roughly to periods of hyperkinesia and hypokinesia, respectively. In contrast to transgenic mice where the mutant *HTT* is randomly inserted into the mouse genome, knock-in mouse models have the CAG expansion inserted into the mouse huntingtin gene, which allows gene expression in its appropriate genomic and protein context (Menalled, 2005). The transgenic rat model of HD (tgHD) carries a truncated huntingtin cDNA fragment with 51 CAG repeats (Von Horsten et al., 2003). The tgHD model and most knock-in mouse models also manifest a slow progression of the HD phenotype.

There is evidence that DA release is reduced in transgenic mouse models in the late stages of the disease, consistent with what is proposed to occur in human HD. There is a progressive reduction in striatal DA levels in both R6/2 and YAC128 mice concomitant with motor abnormalities (Hickey et al., 2002; Johnson et al., 2006; Callahan and Abercrombie, 2011). Furthermore, motorically asymptomatic R6/2 mice show a significant reduction in DA metabolites by 4 weeks of age (Mochel et al., 2011). Deficits in DA levels and/or release have been attributed to either impaired vesicle loading or a reduction in DA reserve pool vesicles available for mobilization (Suzuki et al., 2001; Ortiz et al., 2010). The tgHD rat model displays an increase in striatal DA levels and DA neurons at the early symptomatic stage in two main sources of striatal DA input, the substantia nigra pars compacta and the ventral tegmental area (Jahanshahi et al., 2010). However, these rats also show impaired DA release dynamics, as demonstrated by a reduction in evoked release of DA (Ortiz et al., 2012). Since these results from animal models are not entirely consistent, future studies on DA release dynamics in HD will be needed to parse out changes in DA levels that occur in the early and late disease stages (Table 1).

In agreement with analyses of HD patients, striatal D1 and D2 receptors also are compromised in HD mouse models. Striatal D1 and D2 receptor binding is reduced early, with deficiencies in DA signaling seen in R6/2 and R6/1 mice (Cha et al., 1998; Bibb et al., 2000; Ariano et al., 2002; Petersen et al., 2002a). Significant reductions also are seen in mRNA levels of striatal D1 and D2 receptors in late stage YAC128 mice, but not in BACHD mice (Pouladi et al., 2012). It is unclear why these differences occur between the two full-length models.

The traditional view of behavioral abnormalities in HD proposes that hyperkinetic choreic movements in the early stages result from initial dysfunction of D2-enriched indirect pathway MSNs, while hypokinesia during the late stages is a consequence of further defects in D1-enriched direct pathway MSNs (Spektor et al., 2002). This view has been challenged by recent data obtained in experimental mouse models of HD (YAC128 and BACHD) crossed with mice expressing EGFP in direct and indirect pathway neurons. In the early hyperkinetic stage (1.5 months of age), direct pathway MSNs receive more excitatory inputs than control animals, whereas indirect pathway MSNs are not as affected. In contrast, in the late hypokinetic stage (12 months of age) both pathways receive less excitatory inputs compared to controls (André et al., 2011b; Galvan et al., 2012).

DAT dysregulation also may mediate key alterations in DA neurotransmission and behavior in HD mouse models. A marked reduction of DAT immunoreactivity is observed in the striatum of R6/2 mice (Stack et al., 2007). DAT knock-out mice present not only neuropathological but also behavioral hallmarks of HD, i.e., elevated striatal extracellular DA levels, selective MSN degeneration, and locomotor hyperactivity (Giros et al., 1996; Jones et al., 1998; Cyr et al., 2006; Crook and Housman, 2012). Additionally, studies of DAT knock-out mice crossed with a knock-in mouse model of HD demonstrate an increase in stereotypic behavior that emerges at 6 months of age before returning to baseline by 12 months. Wild-type mice crossed with these knock-in HD mice merely demonstrate a similar but less pronounced biphasic pattern of locomotor alteration (Cyr et al., 2006). Thus, it can be concluded that enhanced DA transmission in HD mice exacerbates the behavioral phenotype of the disease.

## DA and synaptic plasticity in HD

Striatal long-term depression (LTD), a long-lasting decrease in the efficacy of GLU synapses, can be induced through high frequency afferent stimulation or sustained postsynaptic membrane depolarization paired with activation of presynaptic metabotropic GLU receptors (Calabresi et al., 1994; Kreitzer and Malenka, 2005). Additionally, acetylcholine and activation of DA D2 and endocannabinoid CB1 receptors is necessary for LTD induction (Wang et al., 2006; Singla et al., 2007). Induction of striatal long-term potentiation (LTP), a long-lasting increase in the efficacy of GLU synapses, requires activation of DA D1, NMDA, and muscarinic acetylcholine receptors (Calabresi et al., 1999; Kerr and Wickens, 2001). LTD is more easily induced in the dorsolateral and caudal striatum while LTP is more prevalent in the dorsomedial and rostral striatum (Partridge et al., 2000; Spencer and Murphy, 2000; Smith et al., 2001).

The 3-nitropropionic acid (3-NP) toxin model shows an increase in NMDA receptor-dependent LTP at cortico-striatal synapses (Akopian et al., 2008). This form of LTP is mediated by D1 receptors and can be reversed by exogenous addition of DA or a D2 receptor agonist. In genetic HD mouse models, DA levels and receptor numbers are altered, resulting in impaired synaptic plasticity. Furthermore, R6/2 mice display a significant reduction in D1-receptor mediated LTP in the striatum (Kung et al., 2007). Impaired LTP in the medial prefrontal cortex of presymptomatic R6/1 mice can be reversed by D1 receptor agonists (Dallerac et al., 2011). Additionally, layer II/III cells in the perirhinal cortex of symptomatic R6/1 mice are unable to support LTD, which may be a result of reductions in D2 receptor activation (Cummings et al., 2006). Paired-pulse profiles, which are measures of short-term plasticity, are aberrant in cortical slices from R6/1 mice. Instead of exhibiting paired-pulse depression seen in control mice, mutants show a more facilitatory profile. Quinpirole, a D2 receptor agonist, produces a profile that resembles age-matched controls and restores LTD (Cummings et al., 2006). Evidence that D1 receptor agonists rescue impaired LTP while D2 receptor agonists rescue impaired LTD show that there is much promise in therapeutics targeting DA modulation of synaptic plasticity. These are functional consequences that hold important implications for ameliorating the cognitive deficits in HD.

As cholinergic transmission and DA are involved in both LTD and LTP, disturbances of the DA-acetylcholine balance in synaptic plasticity could lead to behavioral deficits. In several HD rodent models, LTP does not occur in cholinergic interneurons. As a consequence, MSNs do not display depotentiation, a process induced by low frequency stimulation that leads to reversion of LTP and requires activation of muscarinic receptors (Picconi et al., 2006). This lack of depotentiation may represent a synaptic mechanism for early behavioral abnormalities observed in HD (Picconi et al., 2006).

## DA and GLU receptor interactions in HD

Although it is unknown why MSNs preferentially degenerate in HD, one major hypothesis has been that MSNs are more susceptible to excitotoxicity. This theory posits that an excess of excitatory neurotransmitters such as GLU and/or overactivation of GLU receptors, particularly the NMDA receptor, mediate MSN neurodegeneration. Overactivity of NMDA receptors can induce cell death through sustained neuronal membrane depolarization, unchecked Ca^2+^ influx, and/or mitochondrial dysfunction (Difiglia, 1990; Coyle and Puttfarcken, 1993). In addition, although DA exists in high concentrations in the striatum, studies also suggest a toxic role for DA in which cell death is accelerated through increases in free radical production (Hastings et al., 1996; Jakel and Maragos, 2000; Wersinger et al., 2004; Hastings, 2009). In striatal cultures derived from R6/2 mice, MSNs undergo DA-mediated oxidative stress and apoptosis (Petersen et al., 2001). Further, DAT knock-out mice are hypersensitive to 3-NP striatal damage (Fernagut et al., 2002).

DA and GLU neurotransmission are intimately intertwined. Understanding this interplay could help elucidate the cause of biphasic DA changes in human HD. In animal models of HD, biphasic changes in corticostriatal GLU transmission are characterized by initial increases in GLU synaptic activity followed by later decreases (Klapstein et al., 2001; Cepeda et al., 2003; Joshi et al., 2009; André et al., 2011a). Early increases in GLU are associated with cortical hyperexcitability (Cepeda et al., 2003; Spampanato et al., 2008; Cummings et al., 2009) and loss of D2 receptors contributes to increased synaptic activity. Stimulation of corticostriatal neurons has been shown to activate DA release in the striatum (Nieoullon et al., 1978). In addition, DA neurons that modulate GLU release in the corticostriatal pathway are subject to afferent GLU regulation, which is suggested by the presence of GLU receptors on DA neurons (Meltzer et al., 1997). There is substantial evidence for a direct cortico-nigral projection (Afifi et al., 1974; Kornhuber et al., 1984; Naito and Kita, 1994) and work in rodents demonstrates that this pathway both directly and indirectly regulates the firing pattern of DA neurons (Maurice et al., 1999; Sesack and Carr, 2002). Other studies indicate that stimulation of GLU receptors on DA neurons increases DA release in both the substantia nigra and in DA innervated areas (Mintz et al., 1986; Kalivas et al., 1989; Murase et al., 1993). Thus, if DA neuron firing is regulated by frontal cortical neurons, the activity of which is upregulated in early HD, the biphasic trends of DA levels in early and late human HD may be correlated with the biphasic changes of GLU release by cortical afferents. This indicates that biphasic changes in DA levels during early and late HD parallel changes occurring in GLU transmission.

In forebrain neurons, which receive both DA and GLU input, a diminished signal-to-noise ratio can impair both motor and cognitive functions (Kiyatkin and Rebec, 1996). Furthermore, a reduction in DA diminishes the strength of the GLU signal above background activity (Kiyatkin and Rebec, 1996). Recently, Hong and Rebec (2012) developed a theoretical framework suggesting that inflexibility rather than inconsistency is the more relevant problem to explain changes during aging and neurodegeneration. Dysfunction in the DA and GLU systems restricts their ability to modulate neural noise. With aging and neurodegeneration, the range over which DA and GLU can be modulated is decreased, leading to dysfunctional neuronal communication, increased neural noise, and inflexibility in brain activity (Hong and Rebec, 2012). Increased neural noise is evident in HD, appearing as a decrease in burst activity and a loss of correlation in the firing patterns of pairs of neurons in the striatum of HD mice (Miller et al., 2008). As a consequence, behavioral adaptations in response to environmental challenges are reduced.

DA and GLU signaling pathways can synergistically enhance MSN sensitivity to huntingtin toxicity. Studies demonstrate that this deleterious process occurs through D1 but not D2 receptor activation (Tang et al., 2007; Paoletti et al., 2008) and are in agreement with previous studies demonstrating that DA and D1 receptor agonists enhance excitotoxicity (Cepeda and Levine, 1998; McLaughlin et al., 1998). D1 receptor-mediated potentiation of NMDA responses, which holds key functional consequences in HD, has been verified in the cortex and striatum (Cepeda et al., 1993; Wang and O'donnell, 2001; Flores-Hernandez et al., 2002). For example, D1 receptor-induced cell death in MSNs of knock-in HD mice is increased with pretreatment with NMDA when compared with cells from wild-type mice (Paoletti et al., 2008). In neurons from YAC128 mice or Q111 knock-in mice, the convergence of DA and GLU signaling pathways leads to Ca^2+^ overload, resulting in excitotoxic processes such as induction of mitochondrial depolarization and caspase activation (Cepeda et al., 2001; Zeron et al., 2002, 2004; Tang et al., 2007; Paoletti et al., 2008).

While D1-NMDA receptor activation is thought to be neurotoxic, activation of D2 receptors reduces NMDA receptor responses and thus may be neuroprotective (Lee et al., 2002; Bozzi and Borrelli, 2006; Blanke and Vandongen, 2009). For example, activation of D2 receptors by quinpirole reduces the toxicity of both NMDA and kainic acid in rat striatal neurons (Cepeda and Levine, 1998), as well as in mesencephalic and cortical neurons (Sawada et al., 1998; Kihara et al., 2002). However, an exclusive role for D1 receptor activation in mediating MSN degeneration is contradicted by evidence that blocking D2 receptor stimulation significantly reverses DA potentiation of mutant huntingtin-induced MSN cell death (Charvin et al., 2005). As cultured striatal neurons can be protected by antagonism of D1 and D2 receptors, it is possible that both D1 and D2 receptor activation might contribute to neurotoxicity (Davis et al., 2002; Bozzi and Borrelli, 2006). Thus, the exact nature of DA and NMDA interactions are dynamic and complex, indicating a need for further investigation into the differential effects of D1 and D2 activation on GLU signaling in the HD striatum.

## DA agonists and antagonists as treatments for HD

Since the abnormalities in the DA system appear to underlie many of the behavioral symptoms of HD, DA agonists, antagonists, and/or stabilizers may provide potential treatment options (Table 2). Conceptually, DA stabilizers (or partial agonists) increase or decrease DA receptor activity depending on the level of DA tone. HD patients treated with aripiprazole, a partial D2 receptor agonist, demonstrate improvements in chorea, but not cognitive function (Brusa et al., 2009). A recent phase 3 clinical trial of the DA stabilizer pridopidine demonstrated improvements in hand movements, gait, and balance of HD patients as defined by the unified HD rating scale (De Yebenes et al., 2011). Although these changes fell short of the primary efficacy threshold, the slight improvements in motor dysfunction without any deleterious side effects suggest that treatments targeted toward DA imbalance may have therapeutic benefits.

**Table 2:** **Available and potential treatments**.

**HUMAN HD**	
Tetrabenazine	Well-supported antichoreatic effects but frequent adverse reactions limit its usefulness (Huntington Study Group, 2006).
D2 antagonists	*Haloperidol*: a traditional D2 antagonist; improves chorea, but does not increase functional capacity (Bonelli and Wenning, 2006). *Olanzapine and risperidone*: atypical antipsychotic drugs with D2 antagonist properties; improve chorea and behavioral disturbances (Squitieri et al., 2001; Duff et al., 2008).
D2 agonists	*Bromocriptine*: effects are both positive and negative (Frattola et al., 1977; Caraceni et al., 1980). *Lisuride*: limited positive effects (Caraceni et al., 1980; Frattola et al., 1983). *Aripiprazole*: a partial D2 agonist; improves chorea but not cognitive function (Brusa et al., 2009).
Other DA drugs	*Pridopidine*: a DA stabilizer; produces slight improvements in motor dysfunction (De Yebenes et al., 2011). *L-DOPA*: possibly useful for treatment of rigidity (Racette and Perlmutter, 1998).
**ANIMAL MODELS**	
Tetrabenazine	Alleviates motor alterations and reduces striatal loss in both early and late stages (Tang et al., 2007; Wang and Morris, 2010; André et al., 2011a).
D1 antagonist	*SCH23390*: rescues electrophysiological changes in excitatory and inhibitory synaptic transmission in direct pathway MSNs (André et al., 2011a).
D1 agonist	*SKF38393*: reverses impaired LTP in the medial prefrontal cortex of presymptomatic R6/1 mice (Dallerac et al., 2011).
D2 antagonist	*Haloperidol*: early and chronic treatment significantly reduces striatal toxicity in the tgHD rat model (Charvin et al., 2008).
D2 agonist	*Quinpirole*: restores the ability of transgenic cortical slices to support LTD (Cummings et al., 2006).

Current treatment options for HD are limited and confined to antidopaminergic agents for motor symptoms while there are virtually no therapeutics for cognitive deterioration (Venuto et al., 2012). Additionally, clinical results of these treatments seem contradictory, possibly reflecting the dynamic and time-dependent changes that occur in the DA system as the disease progresses (Mochel et al., 2011). For example, both D2 agonists and antagonists have demonstrated clinical benefits for improvement of HD motor symptoms (Tedroff et al., 1999; Haskins and Harrison, 2000; Brusa et al., 2009). Conventional antipsychotic drugs, such as the D2 antagonist haloperidol, are used in clinical practice, but they do not improve functional capacity (Bonelli and Wenning, 2006). Atypical antipsychotic drugs with D2 antagonist properties such as olanzapine, risperidone, quetiapine, and ziprasidone, can improve chorea and impact a larger range of behavioral disturbances with a reduced risk of side effects (Squitieri et al., 2001; Bonelli et al., 2003; Alpay and Koroshetz, 2006; Duff et al., 2008). D2 agonists like bromocriptine and lisuride have also demonstrated therapeutic potential in HD (Frattola et al., 1977, 1983; Caraceni et al., 1980).

As the early stages of HD may reflect a hyperdopaminergic stage, drugs that reduce DA tone can be beneficial during the choreic movement phase (Mochel et al., 2011). DA-depleting agents such as tetrabenazine (TBZ), which inhibits vesicular monoamine transporter type-2 and decreases DA content in presynaptic vesicles, have been shown to reduce chorea (Huntington Study Group, 2006). Currently, TBZ is the only drug formally approved for treatment of Huntington's chorea by a regulatory agency (Mestre and Ferreira, 2012).

*In vivo* and *in vitro* studies of animal models support a role for DA inhibitors in protecting HD MSNs from cell death. The rationale follows and agrees with experimental and clinical findings suggesting that DA tone is elevated during the early stages of the disease. In YAC128 mice, TBZ alleviates motor deficits and reduces striatal loss in both early and late stages (Tang et al., 2007; Wang et al., 2010). TBZ also rescues the increased stereotypies in 1–2 month old YAC128 and BACHD mice (André et al., 2011a). D1 receptor antagonists rescue the changes in excitatory synaptic transmission of direct pathway MSNs that occur in the early symptomatic phase of YAC128 and BACHD mice, suggesting that tonic activation of D1 receptors may underlie early dysfunction of D1 MSNs (André et al., 2011a). Similarly, D1 receptor antagonists prevent DA/GLU-induced MSN death in YAC128 mice (Tang et al., 2007). In a lentivirus-based rat model, striatal toxicity is reduced by early and chronic treatment with haloperidol (Charvin et al., 2008). However, this evidence is complicated by the fact that haloperidol, a putative D2 receptor antagonist, also modulates NMDA receptor function (Fletcher and Macdonald, 1993; Ilyin et al., 1996; Arvanov et al., 1997). Predictably, DA antagonists may be more beneficial when administered with other neuroprotective drugs such as memantine, a NMDA receptor antagonist, as a combination therapy (Wu et al., 2006).

HD mouse models have demonstrated the therapeutic potential of not only DA antagonists, but also DA agonists. For example, in fully symptomatic R6/2 mice, replacement of reduced DA levels by chronic treatment with L-DOPA yields short-term improvements in the HD behavioral phenotype whereas long-term treatment impairs survival and rotarod performance (Hickey et al., 2002). Additionally, D1 receptor agonists rescue cortical LTP impairment and deficits in synaptic plasticity of R6/1 mice (Dallerac et al., 2011), suggesting that increasing DA levels could improve cognitive dysfunction. Since some treatments may only be suitable early or late in disease progression, effective therapies need to be temporally oriented to accommodate differential changes in DA levels throughout the course of the disease.

## Conclusions and future directions

While the role of DA in Parkinson's disease is well-established, its role in HD is less well-understood. Although an association between chorea and excess DA levels had long been suspected, a causal link was not demonstrated until TBZ was shown to alleviate abnormal movements in HD. Other less known alterations in early symptomatic patients, such as cognitive changes, impulsivity, gambling, and hypersexuality, could also associate with perturbations of the DA system (Fedoroff et al., 1994; Stout et al., 2001; Rosenblatt, 2007; Beglinger et al., 2008; Jhanjee et al., 2011). TBZ can treat chorea and other early symptoms by reducing DA, but it can also have deleterious effects on cognitive function. Understanding time- and region-dependent alterations in DA function throughout the course of the disease will help in discovering better therapeutic strategies. Selective manipulation of DA-producing neurons, such as using optogenetics in animal models and potentially in human patients, may open new and exciting alternatives.

While much knowledge on the role of DA in HD has been gathered in the past few years, many questions remain unanswered and should be the focus of future endeavors. The traditional view that D2 MSNs are more vulnerable in HD is beginning to change due to emerging data from experimental animal models. Based on evidence reviewed here, one may think that, in fact, D1 MSNs should be more vulnerable to the HD mutation, i.e., they become dysfunctional in the early stage of HD and D1-NMDA receptor interactions enhance neurotoxicity. Therefore, the standing question should be reformulated to ask why D1 MSNs are less susceptible in HD. Do they have a neuroprotective mechanism that D2 MSNs lack? Recent studies using mice expressing EGFP in D1 or D2 cells point in that direction. For example, fluorescence-activated cell sorting array analyses showed that the transcription factor Zfp521, which is enriched in D1 MSNs, is anti-apoptotic (Lobo et al., 2008). Specifically, Zfp521 promotes proliferation, delays differentiation, and reduces apoptosis (Shen et al., 2011).

Another important question is: what causes early perturbations in DA release? Is it the loss of striosome MSN projections to the substantia nigra pars compacta, increased activity along the cortico-nigral projection, or dysregulation of DA release due to loss of D2 auto-receptors? On a similar note, since there are at least two splice variants for D2 receptors, a short D2S (mostly presynaptic) and a long D2L (mostly postsynaptic) form, which one is reduced in early HD? In the striatum, DA D2 auto-receptor function is mediated by synapsin III, a phosphoprotein that is specifically involved in regulating vesicular reserve pools and DA release in the striatum (Feng et al., 2002; Kile et al., 2010). In brains of R6/2 mice and HD patients, there is a progressive loss of complexins, synaptic proteins similar to syntaxin III that are involved in synaptogenesis and modulate neurotransmitter release (Freeman and Morton, 2004). If a similar reduction in synapsin III occurs, this could explain increased DA transmission in early HD and a consequent loss of behavioral flexibility. In agreement, reversal learning can be improved by increasing levels of synapsin III (Laughlin et al., 2011). Thus far, it is unknown whether or not presynaptic D2 auto- or hetero-receptors are lost before postsynaptic receptors (Sandstrom et al., 2010). However, selective agonists of D2 auto-receptors produce long-lasting suppression of extracellular brain DA levels *in vivo* and could provide promising therapeutic benefits for HD (Pifl et al., 1988).

As shown in this review, our knowledge of changes in DA function in HD has made substantial strides, particularly after the introduction of genetic rodent models. However, many more questions remain. Answering these questions is within reach and use of these animal models should help understand the early mechanisms of striatal DA dysfunction and its role in behavioral alterations.

### Conflict of interest statement

The authors declare that the research was conducted in the absence of any commercial or financial relationships that could be construed as a potential conflict of interest.
